# Clinical implementation of accelerated T_2_ mapping: Quantitative magnetic resonance imaging as a biomarker for annular tear and lumbar disc herniation

**DOI:** 10.1007/s00330-020-07538-6

**Published:** 2020-12-03

**Authors:** Marcus Raudner, Markus M. Schreiner, Tom Hilbert, Tobias Kober, Michael Weber, Anna Szelényi, Reinhard Windhager, Vladimir Juras, Siegfried Trattnig

**Affiliations:** 1 Department of Biomedical Imaging and Image-guided Therapy, High Field MR Center, Medical University of Vienna, Lazarettgasse 14, 1090 Vienna, Austria; 2 Christian Doppler Laboratory for Clinical Molecular MR Imaging (MOLIMA), Department of Biomedical Imaging and Image-guided Therapy, High Field MR Center, Medical University of Vienna, Vienna, Austria; 3 Department of Orthopaedics and Trauma Surgery, Medical University of Vienna, Vienna, Austria; 4 Advanced Clinical Imaging Technology, Siemens Healthcare, Lausanne, Switzerland; 5 Department of Radiology, Lausanne University Hospital and University of Lausanne, Lausanne, Switzerland; 6 LTS5, Ecole Polytechnique Fédérale de Lausanne (EPFL), Lausanne, Switzerland; 7 Department of Imaging Methods, Institute of Measurement Science, Bratislava, Slovakia

**Keywords:** Spine, Intervertebral disc, Intervertebral disc displacement, Intervertebral disc degeneration

## Abstract

**Objectives:**

This study evaluates GRAPPATINI, an accelerated T_2_ mapping sequence combining undersampling and model-based reconstruction to facilitate the clinical implementation of T_2_ mapping of the lumbar intervertebral disc.

**Methods:**

Fifty-eight individuals (26 females, 32 males, age 23.3 ± 8.0 years) were prospectively examined at 3 T. This cohort study consisted of 19 patients, 20 rowers, and 19 volunteers. GRAPPATINI was conducted with the same parameters as a conventional 2D multi-echo spin-echo (MESE) sequence in 02:27 min instead of 13:18 min. Additional T_2_ maps were calculated after discarding the first echo (T_2-WO1ST_) and only using even echoes (T_2-EVEN_). Segmentation was done on the four most central slices. The resulting T_2_ values were compared for all four measurements.

**Results:**

T_2-GRAPPATINI_, T_2-MESE_, T_2-EVEN_, and T_2-WO1ST_ of the nucleus pulposus of normal discs differed significantly from those of bulging discs or herniated discs (all *p* < 0.001). For the posterior annular region, only T_2-GRAPPATINI_ showed a significant difference (*p* = 0.011) between normal and herniated discs. There was a significant difference between T_2-GRAPPATINI_, T_2-MESE_, T_2-EVEN_, and T_2-WO1ST_ of discs with and without an annular tear for the nucleus pulposus (all *p* < 0.001). The nucleus pulposus’ T_2_ at different degeneration states showed significant differences between all group comparisons of Pfirrmann grades for T_2-GRAPPATINI_ (*p* = 0.000–0.018), T_2-MESE_ (*p* = 0.000–0.015), T_2-EVEN_ (*p* = 0.000–0.019), and T_2-WO1ST_ (*p* = 0.000–0.015).

**Conclusions:**

GRAPPATINI facilitates the use of T_2_ values as quantitative imaging biomarkers to detect disc pathologies such as degeneration, lumbar disc herniation, and annular tears while simultaneously shortening the acquisition time from 13:18 to 2:27 min.

**Key Points:**

*• T*_*2-GRAPPATINI*_*, T*_*2-MESE*_*, T*_*2-EVEN*_*, and T*_*2-WO1ST*_ *of the nucleus pulposus of normal discs differed significantly from those of discs with bulging or herniation (all p < 0.001).*

*• The investigated T*_*2*_ *mapping techniques differed significantly in discs with and without annular tearing (all p < 0.001).*

*• The nucleus pulposus’ T*_*2*_ *showed significant differences between different stages of degeneration in all group comparisons for T*_*2-GRAPPATINI*_ *(p = 0.000–0.018), T*_*2-MESE*_ *(p = 0.000–0.015), T*_*2-EVEN*_ *(p = 0.000–0.019), and T*_*2-WO1ST*_ *(p = 0.000–0.015).*

## Introduction

Lower back pain (LBP) still ranks as the most common cause for years lived with disability (YLD) and disability-adjusted life years (DALY) according to the Global Burden of Disease Studies of 2016 [1].

The intervertebral disc (IVD) is mainly composed of an inherent architecture of dense collagen fibers, the annulus fibrosus, surrounding the nucleus pulposus with its high concentration of glycosaminoglycans (GAG) that facilitate its water-storing capabilities [2].

Degenerative disc disease, caused by an irreversible decay of the IVD structural integrity, leads to an inadequate biomechanical response to pressure or load [3]. This puts patients at risk for disc herniation, annular tear, or osteochondrosis and leads to chronic pain which is further aggravated by the visceral pain qualities of chronic lower back pain caused by the sinuvertebral nerve endings that sprout into the disc [4].

Visceral pain aggravates with distension and is highly responsive to inflammatory stimuli. It is difficult to localize, while the pain is often described as profound, pressing, and blunt.

To assess the intervertebral disc, non-invasive MRI established itself as the method of choice [5].

Using conventional magnetic resonance imaging (MRI), intervertebral disc degeneration is typically scored using the Pfirrmann classification [6].

However, this presumably simple task is only semi-quantitative, depends on weighted contrast images, is evaluator-dependent, and comes with pitfalls such as magnetization transfer effects, which can cause a lower signal intensity in the nucleus pulposus, resulting in a lower signal intensity and therefore a lower Pfirrmann classification.

Quantitative magnetic resonance imaging (qMRI) methods such as T_2_ mapping provide objective results, which have already shown excellent discriminability when it comes to disc degeneration [7].

As T_2_ mapping is widely available and well-suited for the evaluation of the disc’s biochemical state, correlating with histology, water content, and degeneration is one of the most often used quantitative methods in research regarding low back pain [8].

Also, T_2_ mapping can depict changes in very early stages of disc degeneration that remain invisible to conventional morphological imaging sequences [9].

T_2_ mapping of the IVD can therefore be considered an important biomarker in clinical applications [10, 11].

Conventional T_2_ mapping is usually performed using a two-dimensional multi-echo spin-echo (2D-MESE) sequence which samples multiple contrasts at successive, equally spaced echo times (TE) [12]. The resulting T_2_ maps are reconstructed by performing a mono-exponential voxel-wise fit on the measured signal decay. However, the clinical feasibility of the MESE sequence is limited due to long acquisition times and specific absorption rate (SAR) limitations. These limitations become especially restrictive at a higher in-plane resolution (e.g., 1 × 1 mm^2^) [13].

To overcome these obstacles, GRAPPATINI combines “model-based accelerated relaxometry by iterative non-linear inversion” (MARTINI) and “generalized autocalibrating partial parallel acquisition” (GRAPPA) [14]. GRAPPATINI uses Cartesian sampling with an undersampling pattern organized in blocks based on the chosen undersampling factor. Also, GRAPPA is used for further acceleration by sampling only every other phase-encoding step within a block. The fitting, which is integrated in the image reconstruction, discards the first echo as it is commonly done for MESE data. GRAPPATINI has been successfully applied in other studies with continuously robust T_2_ values compared to a typical MESE sequence in MRI of the brain, knee, prostate, and liver [14].

The aim of this study was (a) to investigate whether GRAPPATINI can be used in direct comparison with T_2_ maps reconstructed from a conventional MESE sequence dataset, (b) to discriminate between different degeneration states, reflected by Pfirrmann classifications, (c) to differentiate discs with and without herniation, and (d) to distinguish between discs with and without annular tear.

## Methods and materials

### Patients and study design

Upon approval by the institutional review board, 60 individuals were prospectively enrolled in this prospective cohort study. Initially, 20 patients, 20 healthy volunteers, and 20 professional rowers were registered between January 2017 and November 2018. The rowers were included in the study to assess early degenerative changes. One patient aborted the measurement due to pain in prolonged supine position and one healthy volunteer was excluded due to motion artifacts in all sequences. In total, 58 MRIs were evaluated (26 females, 32 males, mean age 23.3 ± 8.1 years; ranging from 16 to 50 years) from 19 patients (9 females, 10 males, mean age 28.3 ± 7.5 years, ranging from 19 to 49 years), 19 volunteers (9 females, 10 males, mean age 21.5 ± 7.3 years, ranging from 17 to 50 years), and 20 rowers (8 females, 12 males, mean age 20.3 ± 7.5 years, ranging from 16 to 50 years). No measurement of the remaining 58 analyzed individuals had to be excluded due to artifacts or other measurement-specific reasons.

Inclusion criteria were 16 to 90 years of age, more than two exercises longer than 1 h per week (rowers only), more than 18 months of training (rowers only), or persistent low back pain (patients only). Exclusion criteria were previous spine surgery, a scoliosis with a Cobb angle of more than 15°, a present oncological diagnosis, a systemic disease affecting bone or cartilage, and claustrophobia.

The study population was chosen specifically to compare young individuals with different degrees of spinal load during everyday life to assess early degenerative changes alongside comparably healthy discs in order to cover a wide spectrum of intervertebral disc states. This rationale seemed adequate to assess the broad applicability of different T_2_ mapping techniques as the results should be comparable in healthy, degenerated, or herniated discs or discs with annular tears as the resulting T_2_ values can be quite different depending on the discs state.

### MRI

MR examinations were performed at a field strength of 3 T (MAGNETOM Prisma^fit^, Siemens Healthcare) with a gradient strength of 80 mT/m, using a 32-channel spine matrix coil in supine position. Individuals received a standard leg support with a maximum height of 15 cm placed under their knees during the measurement. The protocol consisted of the following morphological sequences: sagittal T_1_-weighted TSE, sagittal T_2_-weighted TSE, sagittal T_2_-weighted STIR, and a coronal and axial T_2_-weighted TSE sequence with three sections per intervertebral disc. After the morphological sequences, T_2_ mapping was performed with a MESE sequence and the GRAPPATINI prototype directly afterwards.

A repetition time (TR) of 3500 ms (≥ 3xT_1_, estimated at ≈ 1000 ms) was used in the T_2_ mapping sequences to recover the magnetization in every voxel prior to every echo train [14, 15].

A known caveat in T_2_ mapping is the signal component that arises from stimulated echoes. These are additional signal components which result in an increased and oscillating signal intensity starting from the second echo. Stimulated echoes are caused by suboptimal refocusing pulse angles due to B_1_ field inhomogeneity and imperfect slice profiles. Well-known ways to assure a good and stable fitting in spite of these wrong signal components are either to discard the first echo or to use only even echoes for the T_2_ map reconstruction [16].

Therefore, the echoes acquired with the MESE sequence were used to calculate two additional T_2_ maps directly on the scanner using MapIT (Siemens Healthcare) without the first echo (WO1ST) and using only even echoes (EVEN).

GRAPPATINI was set to an undersampling factor of 5 and two-fold GRAPPA, effectively resulting in ten-fold undersampled k-space data relative to a fully sampled acquisition for the T_2_ map reconstruction. The reference MESE sequence used only a conventional two-fold GRAPPA acceleration. GRAPPATINI T_2_ maps were automatically reconstructed as previously reported [14].

The sequence parameters for all morphological and T_2_ mapping sequences are listed in Table 1.

**Table 1:** Sequence parameters

Parameter	T_2_w TSE	GRAPPATINI	Multi-echo Spin echo
Plane	Sagittal	Sagittal	Sagittal
No. of slices	8	8	8
Slice thickness (mm)	4	4	4
Interslice gap (mm)	0.4 mm	0.4 mm	0.4
Field of view (mm^2^)	260 × 260	260 × 260	260 × 260
Acquisition matrix	384 × 230	378 × 227	384 × 230
Voxel size (mm^3^)	0.7 × 0.7 × 4	0.7 × 0.7 × 4	0.7 × 0.7 × 4
Phase encoding direction	A > > P	A > > P	A > > P
Time to repetition (TR in ms)	3500	3500	3500
Time to echo (TE in ms)	99	9 to 144 ms 16 echoes	9 to 144 ms 16 echoes
No. of averages	1	1	1
GRAPPA	2	2	2
Undersampling factor	–	5	–
Flip angle	160°	180°	180°
Bandwidth (Hz/Px)	224	224	224
Acquisition time	02:01	02:27	13:18

### Image analysis

Morphological images were assessed strictly adhering to the Lumbar Disc Nomenclature 2.0 of Fardon et al [17]. Additionally, all discs were scored after Pfirrmann et al [6].

**Fig. 1:**
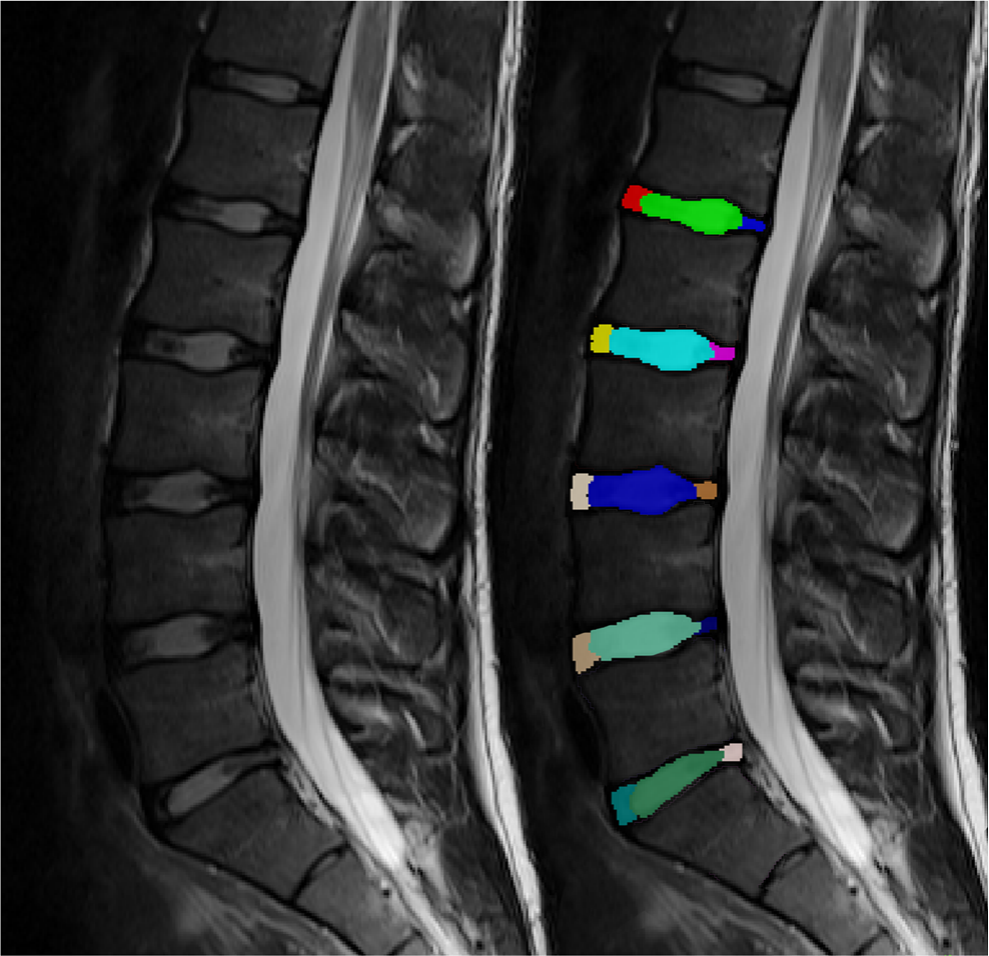
Sagittal T_2_-weighted contrast (left) with a color-coded overlay visualizing the segmentation done in ITK-SNAP (right). Every intervertebral disc was segmented in three individual regions of interest (ROIs): ventral annulus fibrosus, nucleus pulposus, and posterior annulus fibrosus. This case shows an annular tear in L5/S1

The resulting images were manually segmented on the sagittal T_2_-weighted TSE sequence using the four most central slices by a radiology resident with 5 years of experience in musculoskeletal imaging using ITK-Snap [18]. First, a strict geometrical order was followed labeling the anterior and posterior 20% as annulus fibrosus with the central 60% of the diameter representing the nucleus pulposus. This was then manually refined depending on the individual discs. An example is depicted in Fig. 1. Individual labels for each discs’ ventral and posterior annular region and the nucleus pulposus of every of the five lumbar discs were drawn. Using Elastix [19], the segmentation was then copied over to the T_2_ maps after successful automatic co-registration.

### Statistical evaluation

All statistical analyses were performed by a biomedical statistician using IBM SPSS for Windows version 25 (IBM). Given metric and normal distributed data are described using mean ± standard deviation (SD). In case of skewed metric data, median (95% confidence intervals) was used. Correlations were assessed using Spearman’s correlation coefficient. Univariate ANOVA with post hoc tests depending on homogeneity of variances choosing either GT2 after Hochberg (no difference in variance) or Games-Howell (different variance) were used to compare groups (e.g., according to Pfirrmann grading). ROC analyses were used to test for discriminability regarding disc herniation, bulging, and annular tear.

A *p* value of ≤ 0.05 was considered to indicate statistically significant results. In order to avoid an increasing error of the second type, no multiplicity corrections were performed [20].

## Results

### T_2_ mapping methods—GRAPPATINI and conventional MESE

Overall, the nucleus pulposus T_2-GRAPPATINI_ (91.8 ms; CI95 89.2–94.5) showed a very strong Spearman correlation coefficient (all *p* < 0.001; *r* = 0.919 to 9.926) when compared with T_2-MESE_ (104.7 ms; CI95 101.3–108.1) as well as with T_2-EVEN_ (93.3 ms; CI95 90.5–96.2) and T_2-WO1ST_ (94.1 ms; CI95 91.2–97.0). Overall, T_2-MESE_ showed significantly higher T_2_ values in the nucleus pulposus than T_2-GRAPPATINI_, T_2-WO1ST_, and T_2-EVEN_ (all *p* < 0.001).

The posterior annulus fibrosus assessment showed a fair Spearman correlation (all *p* < 0.001; *r* = 0.446 to 0.465) of T_2-GRAPPATINI_ (47.7 ms; CI95 46.8–48.5), T_2-MESE_ (46.6 ms; CI95 45.4–47.8), T_2-EVEN_ (43.4 ms; CI95 42.2–44.5), and T_2-WO1ST_ (44.8 ms; 95CI 43.5–46.0).

Overall, T_2-MESE_ showed significantly higher T_2_ values in the posterior annular region than T_2-WO1ST_ and T_2-EVEN_ (both *p* < 0.001). However, T_2-MESE_ showed insignificantly lower T_2_ compared to T_2-GRAPPATINI_ (*p* = 0.074).

For an overview of all the different assessments and the resulting average median T_2_ values with 95% confidence intervals, see Table 2.

**Table 2:** T_2_ values for each assessment with 95% confidence intervals

Comparison of T_2_ relaxation time measurements				
	T_2-GRAPPATINI_ (CI95)	T_2-MESE_ (CI95)	T_2-EVEN_ (CI95)	T_2-WO1ST_ (CI95)
Lumbar disc herniation				
Nucleus pulposus				
Normal (*n* = 204)	101.3 (98.8–103.7)	116.1 (112.6–119.6)	103.1 (100.3–106.0)	104.0 (101.2–106.9)
Bulging (*n* = 46)	70.9 (64.9–76.9)	81.7 (74.5–88.9)	73.6 (67.4–79.9)	74.2 (67.9–80.4)
Herniation (*n* = 40)	67.8 (63.3–72.3)	72.8 (69.2–76.4)	65.9 (62.7–69.1)	66.4 (63.2–69.6)
Mean (*n* = 290)	91.8 (89.2–94.5)	104.7 (101.3–108.1)	93.3 (90.5–96.2)	94.1 (91.2–97.0)
Annulus fibrosus				
Normal (*n* = 204)	47.0 (46.0–48.0)	45.6 (44.1–47.1)	42.5 (41.0–43.9)	43.9 (42.3–45.5)
Bulging (*n* = 46)	48.1 (45.8–50.4)	48.6 (46.1–51.0)	45.4 (43.1–47.6)	47.0 (44.6–49.5)
Herniation (*n* = 40)	50.7 (48.4–53.1)	49.5 (46.0–53.0)	45.6 (42.5–48.7)	46.7 (43.4–50.0)
Mean (*n* = 290)	47.7 (46.8–48.5)	46.6 (45.4–47.8)	43.4 (42.2–44.5)	44.8 (43.5–46.0)
Annular tear				
Nucleus pulposus				
No tear (*n* = 258)	95.6 (93.1–98.2)	109.2 (105.8–112.6)	97.2 (94.3–100.1)	98.1 (95.2–100.9)
Tear (*n* = 32)	61.0 (57.7–64.3)	68.3 (64.3–72.3)	62.0 (58.5–65.6)	62.3 (58.8–65.9)
Mean (*n* = 290)	91.8 (89.2–94.5)	104.7 (101.3–108.1)	93.3 (90.5–96.2)	94.1 (91.2–97.0)
Annulus fibrosus				
No tear (*n* = 258)	47.4 (46.4–48.3)	45.8 (44.4–46.9)	42.5 (41.3–43.7)	43.9 (42.6–45.2)
Tear (*n* = 32)	50.1 (48.0–52.2)	54.2 (50.2–58.2)	50.4 (46.8–54.0)	51.8 (48.0–55.6)
Mean (*n* = 290)	47.7 (46.8–48.5)	46.6 (45.4–47.8)	43.4 (42.2–44.5)	44.8 (43.5–46.0)
Pfirrmann classification				
Nucleus pulposus				
I (*n* = 151)	104.7 (102.1–107.3)	120.4 (116.5–124.3)	106.8 (103.6–109.9)	107.8 (104.6–110.9)
II (*n* = 55)	97.1 (92.8–101.5)	109.9 (104.3–115.5)	98.4 (93.8–103.1)	99.1 (94.4–103.8)
III (*n* = 35)	73.0 (68.7–77.3)	81.2 (75.8–86.6)	73.3 (68.8–77.9)	73.8 (69.3–78.3)
IV (*n* = 48)	59.7 (57.5–61.8)	67.2 (64.8–69.7)	60.5 (58.4–62.6)	61.1 (59.0–63.2)
V (*n* = 1)	58.3	61.0	53.5	55.0
Annulus fibrosus				
I (*n* = 151)	46.7 (45.5–47.9)	44.6 (43.1–46.2)	41.7 (40.1–43.3)	43.0 (41.3–44.7)
II (*n* = 55)	47.4 (45.1–49.8)	46.7 (43.5–49.8)	43.5 (40.5–46.6)	45.5 (42.0–49.0)
III (*n* = 35)	48.7 (46.5–51.0)	48.2 (45.6–50.9)	44.7 (42.3–47.1)	45.6 (43.2–48.1)
IV (*n* = 49)	50.2 (48.2–52.2)	51.7 (48.3–55.0)	47.6 (44.7–50.4)	49.0 (46.0–51.9)
V (*n* = 0)	47.0	43.5	39.0	39.5

### Lumbar disc herniation assessment

Of all evaluated 290 intervertebral discs, 46 were labeled as bulging discs in 29 individuals and 40 showed lumbar disc herniation in 25 individuals.

T_2-GRAPPATINI_, T_2-MESE_, T_2-EVEN_, and T_2-WO1ST_ of the nucleus pulposus of normal discs differed significantly from those of bulging discs or herniated discs (*p* < 0.001). There was no significant difference between bulging discs or herniated discs for T_2-GRAPPATINI_ (*p* = 0.682), T_2-MESE_ (*p* = 0.072), T_2-EVEN_ (*p* = 0.076), or T_2-WO1ST_ (*p* = 0.075).

**Fig. 2:**
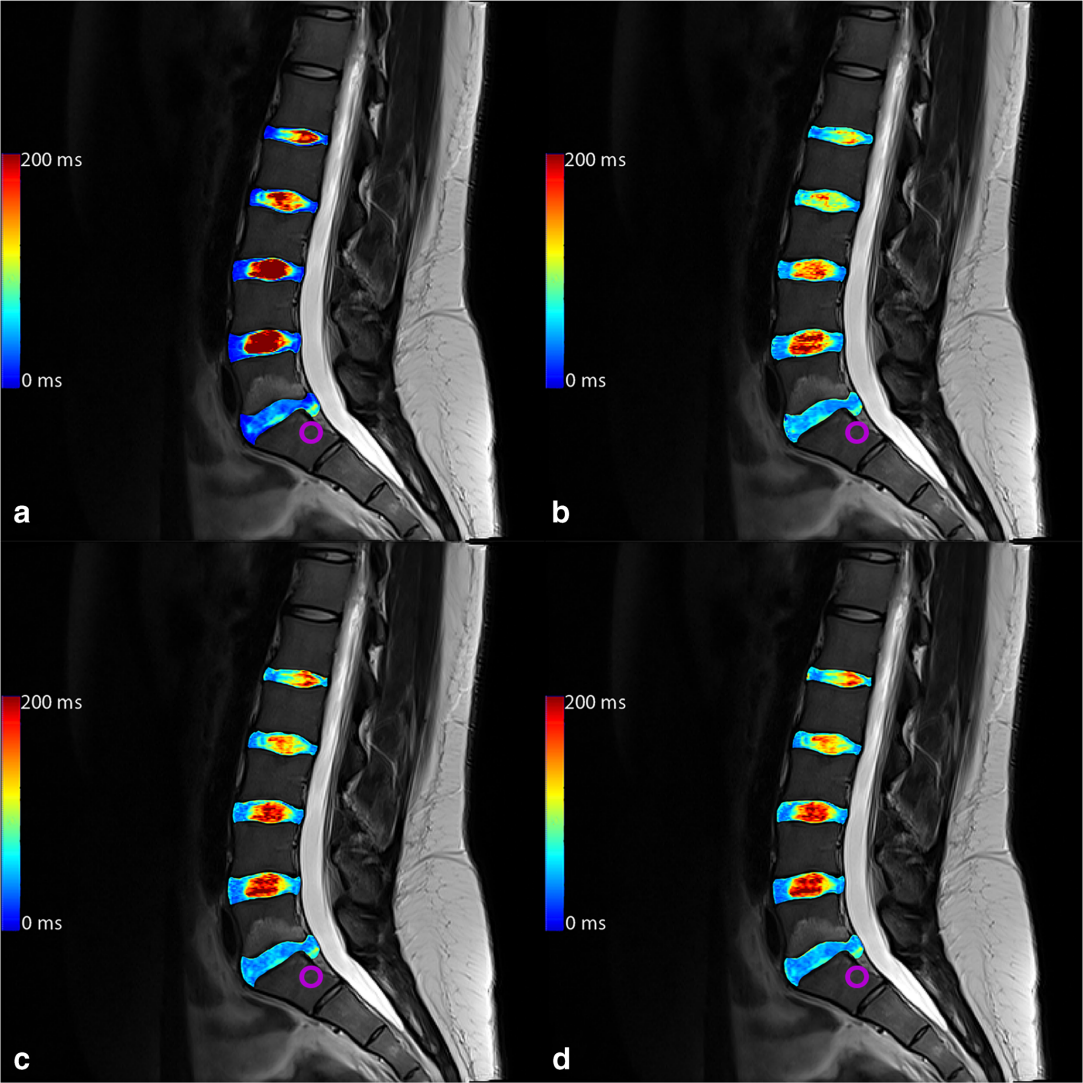
Color-coded T_2_ map overlays of (**a**) multi-echo spin-echo (MESE), (**b**) GRAPPATINI, (**c**) EVEN, and (**d**) WO1ST. The purple circle marks a herniating L5/S1 disc with annular tear with nucleus pulposus tissue in the lower posterior annular region of interest, resulting in higher, pathological T_2_ at that very place and a consecutive acute bone marrow edema in L5

For the posterior annular region, only T_2-GRAPPATINI_ showed a significant difference (*p* = 0.011) between normal and herniated discs. All other group comparisons of T_2-GRAPPATINI_, T_2-MESE_, T_2-EVEN_, and T_2-WO1ST_ showed non-significant results (*p* = 0.086 to 0.999). An example of a patient with herniation in segment L5/S1 is given in Fig. 2 with color-coded T_2_ map overlays.

Calculating a receiver operating characteristic (ROC) assessing healthy discs with neither herniation nor bulging present using the NP T_2_, the area under the curve (AUC) was 0.895 (0.851–0.939) for T_2-GRAPPATINI_, 0.888 (0.844–0.931) for T_2-MESE_, 0.888 (0.844–0.931) for T_2-EVEN_, and 0.890 (0.847–0.933) for T_2-WO1ST_.

Calculating an ROC for bulging discs, the AUC was 0.810 (0.742–0.878) for T_2-GRAPPATINI_, 0.774 (0.700–0.849) for T_2-MESE_, 0.775 (0.700–0.849) for T_2-EVEN_, and 0.776 (0.702–0.850) for T_2-WO1ST_.

Calculating a receiver operating characteristic (ROC) assessing disc herniation using the NP T_2_, the area under the curve (AUC) was 0.845 (0.787–0.904) for T_2GRAPPATINI_, 0.872 (0.829–0.916) for median T_2MESE_, 0.872 (0.829–0.915) for median T_2EVEN_, and 0.874 (0.831–0.917) for median T_2WO1ST_.

### High-intensity zone and annular tear assessment

Of all 290 intervertebral discs assessed, 32 showed a high-intensity zone in 22 individuals in the posterior annular region, indicating annular tearing.

There was a significant difference between T_2-GRAPPATINI_, T_2-MESE_, T_2-EVEN_, and T_2-WO1ST_ of discs with and without HIZ for both the nucleus pulposus and the posterior annular region (all *p* < 0.001).

There was one exception with the posterior annular region closely missing the level of significance in the T_2-GRAPPATINI_ assessment (*p* = 0.052).

**Fig. 3:**
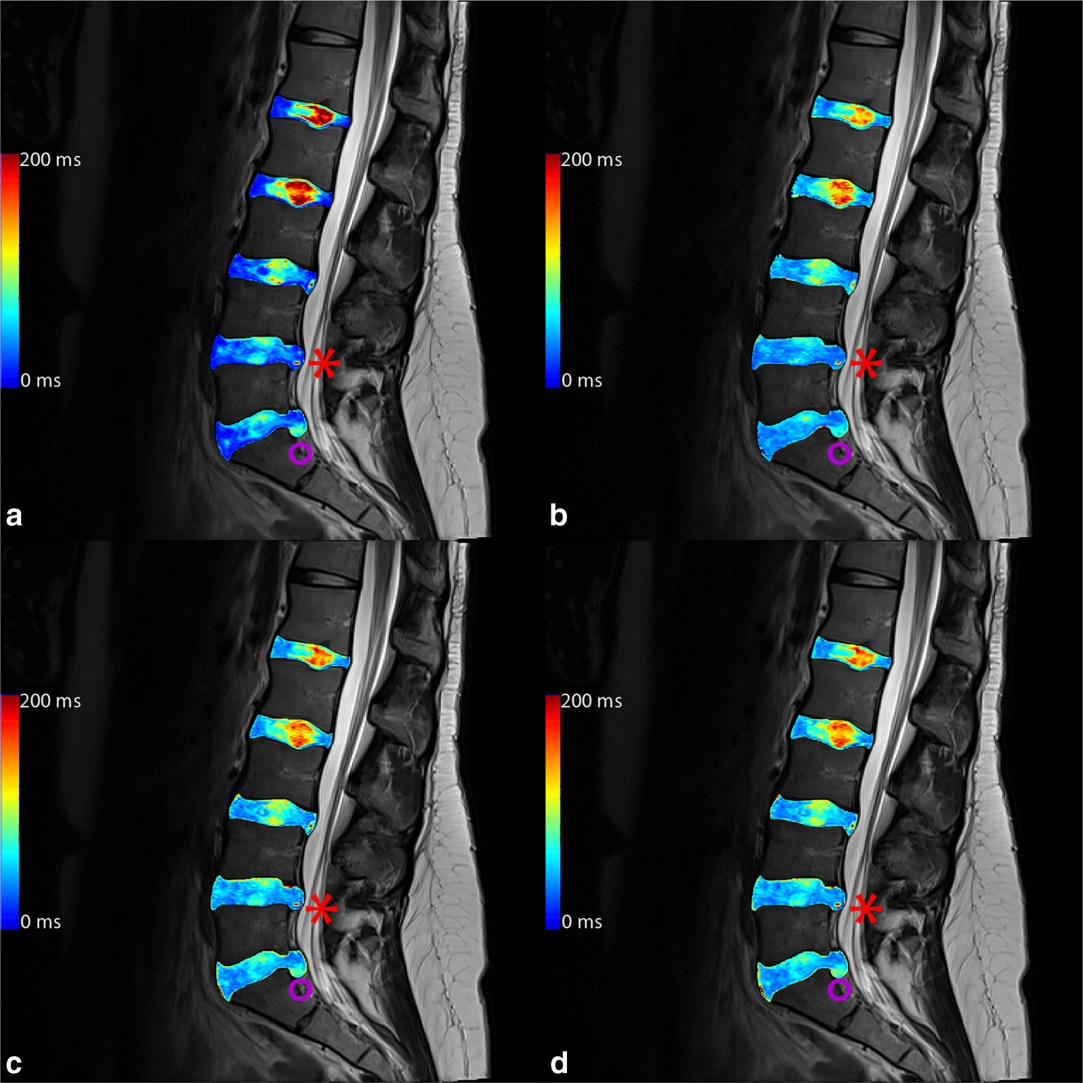
Color-coded T_2_ map overlays of **a** multi-echo spin-echo (MESE), **b** GRAPPATINI, **c** EVEN, and **d** WO1ST. The red asterisk marks a high-intensity zone in L4/L5 which can be clearly seen in all measurements (another small one tear can be seen on L3/L4 also). The purple circle marks a herniating L5/S1 disc with annular tear with nucleus pulposus tissue in the lower posterior annular region of interest, resulting in higher, pathological T_2_ at that very place

Calculating an ROC for HIZ for the nucleus pulposus T_2_, the AUC was 0.919 (0.886–0.953) for T_2-GRAPPATINI_, 0.913 (0.873–0.953) for T_2-MESE_, 0.911 (0.871–0.950) for T_2-EVEN_, and 0.915 (0.876–0.954) for T_2-WO1ST_. An example patient with a combination of lumbar disc herniation and annular tear of the L5/S1 segment alongside a HIZ in the L4/L5 segment is given in Fig. 3 with color-coded T_2_ map overlays.

### T_2_ value differences for respective Pfirrmann grades

Of all 290 discs, the Pfirrmann grades were distributed as follows: I, 151 IVDs; II, 55 IVDs; III, 35 IVDs; IV, 48 IVDs; and V, 1.

For the NP, there was a significant difference between all group comparisons of Pfirrmann grades for T_2-GRAPPATINI_ (*p* value 0.000 to 0.018), T_2-MESE_ (0.000 to 0.015), T_2-EVEN_ (0.000 to 0.019), and T_2-WO1ST_ (0.000 to 0.015).

The annular region showed less discriminability between Pfirrmann grades for T_2GRAPPATINI_ (*p* value 0.027 to 0.991), T_2-MESE_ (0.000 to 0.979), T_2-EVEN_ (0.002 to 0.995), and T_2WO1ST_ (0.005 to 1.000).

## Discussion

Our data show that GRAPPATINI is capable of significantly shortening the acquisition time needed for accurate T_2_ mapping from 13:18 to 2:27 min while maintaining the known advantages of quantitatively reflecting the most common disc pathologies.

T_2-GRAPPATINI_ of the nucleus pulposus showed an AUC comparable to the other methods when assessing discs with neither bulging nor herniation present. The same was true when assessing discs for bulging. Additionally, T_2-GRAPPATINI_ showed the only significant difference in the posterior annular region for healthy discs compared to discs with herniation present. Furthermore, T_2-GRAPPATINI_ showed an AUC equally high as the other methods for HIZ present in the annulus fibrosus.

Over a decade ago, it has already been demonstrated by Perry et al and Watanabe et al that T_2_ mapping may be employed to assess the ultrastructural composition of intervertebral discs, namely highly hydrated glycosaminoglycans (GAG) in the nucleus pulposus and dense collagen fibers in the area of the annulus fibrosus [9, 21].

With ongoing degeneration, the IVD GAG content decreases, leading to a falling swelling pressure and an increased risk for annular tears and disc herniation [2, 22].

Since allegedly simple tasks, such as the semiquantitative Pfirrmann classification [6] for degeneration assessment, can also be prone to error due to magnetization transfer effects or observer bias, quantitative data are desirable to obtain more objective results. T_2_ mapping can do that with similar or better discriminability when it comes to disc degeneration [7, 23].

In line with this statement, Schultz et al reported that the biomechanical properties of the intervertebral discs are better correlated with the collagen fiber structure integrity than with the Pfirrmann classification [24]. Also, Marinelli et al showed that T_2_ mapping correlated with the water and proteoglycan content of the nucleus pulposus in a histological specimen study of calf and human discs [11].

Ogon et al even showed significant differences in the posterior annular region with lower T_2_ values in patients with chronic lower back pain compared to healthy controls [8].

However, even after multiple promising studies, T_2_ mapping of hardly any cartilaginous tissue has found its way into clinical routine mainly due to the clinically unfeasible measurement times.

Our data confirm the aforementioned literature with significant differences of T_2_ values between the different grades of disc degeneration. However, GRAPPATINI was acquired in just 02:28 min with preserved spatial resolution. Furthermore, no sequence-specific artifacts were observed that would impede assessment of an intervertebral disc. This facilitates T_2_ mapping of the entire lumbar spine in 02:28 min as opposed to 13:18 min using a conventional MESE sequence with the same sequence parameters.

Lumbar disc herniation is commonly found in the general population and frequently asymptomatic. However, the presence of lumbar disc herniation is an important finding in symptomatic patients as it significantly worsens prognosis [25]. Hoppe et al have already shown that T_2_ values significantly differ between herniated and non-herniated discs [26]. In a 5-year follow-up study, the baseline T_2_ values of the nucleus pulposus could be used as a predictive biomarker for new lumbar disc herniation at follow-up [27].

Our data are coherent with the literature with significant T_2_ differences in normal discs compared to herniated discs with a similar performance for the investigated T_2_ mapping techniques as measured by the AUCs of calculated ROCs. However, T_2-GRAPPATINI_ was the only assessment showing significant differences in the posterior annular region of herniated compared to normal discs.

Another important finding in general morphological imaging of the lumbar IVDs is the high-intensity zones of the posterior annulus fibrosus [17].

They are caused by torn fibro-cartilage lamellae of the annulus fibrosus containing trapped nucleus pulposus tissue. This promotes the ingrowth of vascularized granulation tissue into the disc. Simultaneously, sinuvertebral nerve endings begin to sprout into the disc which attribute to the visceral pain qualities of chronic low back pain [4].

In this study, discs with HIZ showed significantly different T_2_ values in the nucleus pulposus and the posterior annular region compared to discs without HIZs with T_2-GRAPPATINI_ of the nucleus pulposus showing an AUC comparable to the other T_2_ mapping methods when testing for discriminability.

This is concordant with the findings of other studies that reported measurable differences in discs with and without HIZ using T_2_ mapping [28].

However, groups like Sharma et al have stated that annular tears precede and accelerate—or actually cause—disc degeneration rather than mainly occurring in already degenerated discs [29]. Our data suggest the contrary, hinting more at annular tear occurring earlier than the associated disc degeneration, as this study’s participants were very young. However, this has to be proven by larger prospective or longitudinal case-control studies, which could be facilitated by accelerated T_2_ mapping sequences like GRAPPATINI.

Another noteworthy finding in the presented data is the significantly higher T_2-MESE_ of the nucleus pulposus when compared to T_2-GRAPPATINI_, T_2-EVEN_, and T_2-WO1ST_. This overestimation is mainly caused by stimulated echoes which instigate an increased signal proportion along the echo train. This results in an overestimated T_2_ relaxation time and unstable fitting procedure. The fitting can, however, be substantially improved by calculating the T_2_ maps without the first or with only even echoes, as reflected in T_2-WO1ST_ and T_2-EVEN_. This approach to mitigate bias caused by stimulated echoes has already been used in other studies [14, 16], however still results in a small systemic overestimation of T_2_.

Additionally, robust T_2_ mapping has been a challenging field for many years already due to imperfect slice profiles, field inhomogeneities, and flip angle offsets [30].

There are other attempts to shorten the acquisition time needed for T_2_ relaxation time measurements, like radial T_2_ mapping sequences, the three-dimensional triple-echo steady-state sequence, or, in a much broader perspective, magnetic resonance fingerprinting (MRF) [31–33]. However, none of the aforementioned has been effectively used in T_2_ mapping of the lumbar intervertebral disc to this date. This can be attributed mostly to the heterogeneity in the field of view, significant pulsation artifacts from the aorta, and the challenging distance combined with the narrow potential for parallel imaging using spine coils [32].

Also, MRF remains a promising, but investigational approach with ongoing dispute considering the generation of used dictionaries, image reconstructions requiring unusual computational resources, and the currently restricted in-plane resolution in clinically feasible acquisition times.

This study has some limitations to be addressed.

First, GRAPPATINI requires a long echo train since it uses the information along the echoes to recover non-sampled k-space lines. Consequently, the final protocol had to include 16 echoes (TE_max_ = 144 ms) for an undersampling factor of 5, which may cause the fitting of a noise plateau in the calculated T_2_ decay for voxels with shorter relaxation times. However, this can be relativized by the annular T_2_ values which still show significant differences for the assessed pathologies at relatively short T_2_ times which were not superimposed by a noise component.

Also, the studied individuals of this study were comparably young. This, however, was intended, as the idea was to assess early stages of degeneration and pathology. In the end, there were enough cases of already degenerated or herniated discs to allow for a sufficient analysis.

Additionally, the segmentation used for the T_2_ value assessment was done by one single reader who was blinded to any clinical information. The segmentation was, however, copied from the T_2_w TSE images to the resampled T_2_ maps, so there was no observer-based difference in the respective quantitative sequence comparisons.

In conclusion, the presented study shows that “generalized autocalibrating partially parallel acquisition and model-based accelerated relaxometry by iterative non-linear inversion,” in short GRAPPATINI, facilitates robust T_2_ mapping with a ten-fold reduction of measurement time (2:27 min) with the potential to use the resulting T_2_ maps as an imaging biomarker for disc degeneration, annular tear, and herniation. At the same time, T_2-GRAPPATINI_ showed an equal or better performance when directly compared to T_2-MESE_, T_2-EVEN_, and T_2-WO1ST_.

## Abbreviations

2D-MESE: Two-dimensional multi-echo spin-echo sequence
3D-TESS: Three-dimensional triple-echo steady-state sequence
CI95: 95% confidence interval
CPMG: Carr-Purcell-Meiboom-Gill sequence
ERL: Echo train length
EVEN: Reconstructed T_2_ maps based on MESE data based on only even echoes
GRAPPA: Generalized autocalibrating partial parallel acquisition
GRAPPATINI: Sequence combining MARTINI and GRAPPA
MARTINI: Model-based accelerated relaxometry by iterative non-linear inversion
MRF: Magnetic resonance fingerprinting
qMRI: Quantitative magnetic resonance imaging
SAR: Specific absorption rate
SD: Standard deviation
TE: Time to echo (in milliseconds)
TR: Time to repetition (in ms if not otherwise stated)
WO1ST: Reconstructed T_2_ maps based on MESE data excluding the first echo
